# Pazopanib Selectively Inhibits Choroidal Vascular Endothelial Cell Proliferation and Promotes Apoptosis

**Published:** 2019-10-30

**Authors:** Bharani Mynampati, Moises Enghelberg, Kakarla V. Chalam

**Affiliations:** 1 Department of Ophthalmology, Loma Linda University School of Medicine, Loma Linda, California, USA

**Keywords:** Pazopanib, In vitro, Age Related Macular Degeneration, Vacular Endothelial Growth Factor, Choroidal Vascular Endothelial Cell, Bevacizumab, Ranibizumab, Aflibercept

## Abstract

Exudative age related macular degeneration (AMD) is related to active choroidal neovascularization (CNV) and formation of disciform scars. Vascular endothelial growth factor (VEGF) mediated choroidal vascular endothelial cell (CVECs) proliferation is characteristic of CNV. Intravitreal injections of bevacizumab, ranibizumab and aflibercept (anti-VEGF monoclonal antibodies) are used to treat exudative AMD. Pazopanib, a tyrosine kinase inhibitor, inhibits neovascularization through blockade of intracellular tyrosine kinase VEGF receptor and platelet-derived growth factor receptor. In this *in vitro* investigation, we evaluated the inhibitory consequences of escalating doses of pazopanib on proliferation of VEGF-enriched CVECs to establish a safe dosage range. VEGF **(**50 ng/mL) enriched CVECs were treated with escalating doses of pazopanib (10, 50,100 and 250 µM). Cell proliferation rates (WST-1 assay), cell viability (trypan blue exclusion assay), and reactive oxygen species (ROS) levels were measured at 48 hours (h), 72h and 1 week. Intracellular caspase 3 levels and morphological changes were recorded. VEGF enriched CVECs showed a significant decrease in cell proliferation rates after one week of treatment with increasing doses of pazopanib (10, 50,100 and 250 µM) treatment i.e. 87.8%, 43.0%, 38.1% and 9.3% compared to controls (p<0.001). Similarly, trypan blue exclusion assay revealed a decrease in cell viability as 81.8%, 81.0%, 53.4% and 8.7%, respectively (p<0.05). Further, pazopanib actively inhibited proliferation of VEGF-enriched CVECs, with 1.32, 1.92, 1.92 and 4.1-fold increase (p<0.01) in intracellular caspase 3 levels. VEGF-enriched CVECs treated with escalating doses of pazopanib decreased cell viability and increased caspase 3 levels in a time and dose dependent manner.

## INTRODUCTION

Age-related macular degeneration (AMD) is the most common retinal cause of visual impairment in the United States at present [1]. Neovascular (exudative) AMD, is a less common condition but with more advanced and aggressive form of AMD, which affects 15% of cases and involves active choroidal neovascularization (CNV) and formation of disciform scar at late stages [2]. Pathophysiology of CNV is characterized by vascular endothelial growth factor (VEGF) mediated choroidal vascular endothelial cell (CVECs) proliferation, a potential target for antivascular endothelial growth factor therapy (anti-VEGF) [3].In the past, CNV associated with AMD was treated by transferring thermal energy to the proliferating CVECs with conventional or modified laser (photodynamic therapy) [4]. Nevertheless, thermal energy lacked the differential tissue sensitivity and destroyed the proliferating CVECs together with the overlying retina. Loss of stable CVECs may lead to further ischemia, consecutive upregulation of VEGF and consequences in recurrent proliferation of abnormal CVECs [5].

The imbalance between proangiogenic and antiangiogenic factors has been associated with a number of severe eye diseases such as neovascular AMD, diabetic retinopathy, sickle cell retinopathy and retinopathy of prematurity [6-9]. Although neovascularization is a complex process involving endothelial cell proliferation, migration and tissue degradation, VEGF inhibition is sufficient to inhibit CNV. Accordingly, VEGF inhibitors has been used to control neovascularization in pathological conditions such as cancer [10], retinopathy of prematurity [11] and neovascular AMD [12].

Bevacizumab intravitreal injection as an anti-VEGF monoclonal antibody is effective in the treatment of exudative AMD. Ranibizumab and Aflibercept are other anti-VEGF drugs used in the treatment of various ocular diseases [13]. Newer and experimental medications such as Brolucizumab and Abicipar (single-chain antibody fragment) are currently in Phase 3 trials and may complement the treatment of neovascular AMD [14-18].

Pazopanib, a tyrosine kinase inhibitor (TKI), (FDA approved drug for advanced or metastatic renal cell carcinoma), inhibits neovascularization through blockade of intra-cellular tyrosine kinase VEGF receptor and platelet-derived growth factor receptor (PDGFR) [19]. This makes pazopanib an attractive and compelling alternative for consideration in the treatment of neovascular AMD. Preliminary clinical studies establishing the efficacy of pazopanib in the management of CNV is encouraging [19]. However, the dose and mechanism of action of pazopanib is empiric.

In this *in vitro* study, we studied the inhibitory effects of escalating doses of pazopanib on the VEGF-enriched CVECs proliferation to establish a safe dose range.

## METHODS

**Cell culture**

Ethical approval for this study was received from our academic institution. Choroidal vascular endothelial cells (CVECs) (RF/6A) were taken from the American Type Culture Collection (ATCC-Manassas, VA. # CRL-1780) and cultured in Eagle’s minimal essential medium (EMEM; Invitrogen, Carlsbad, CA, The USA) comprising 10% fetal bovine serum (FBS; Sigma, St. Louis, MO, The USA), 100 units/mL penicillin, and 100 microgram per millilitre (μg/mL) streptomycin. These choroidal cells were retained in log phase growth. Cells were harvested at 37 °C in logarithmic scale in 75 Square Centimeter cell culture flasks.

**Enrichment of choroidal vascular endothelial cells with VEGF**

CVECs were treated with 50 nanogram/milliliter (ng/mL) doses of human VEGF165 (Pepro Tech, Rocky Hill, NJ, The USA) to keep cells in stable optimal proliferative mitotic stage and imitate human disease processes (sub-retinal neovascular membrane [SRNVM]) and proliferative diabetic retinopathy (PDR). Successive experiments were conducted employing CVECs enriched for 48 hours using VEGF (50 ng/mL) [6].

**Treatment of CVECs with Pazopanib**

CVECs were treated with increasing doses of pazopanib (Santa Cruz, The USA) at concentrations of 10, 50, 100 and 250 µM. The exposure was maintained up to a maximum period of one week and cellular activity was re-evaluated at different time points (48h, 72h and 1 week) for all pazopanib concentrations. Cell viability was compared to control cells not treated with pazopanib. Tested and control cells were surveyed at the same time points.

**Assessment of Cellular Viability**

**Cell proliferation using WST-1 Assay**

VEGF enriched CVECs were plated at a density of 20,000 cells/well in 96 well plates for 48h and then exposed to different concentrations of pazopanib as aforementioned. Cellular growth was re-evaluated in accordance with the manufacturer’s guidelines with the 4-[3-(4-lodophenyl)-2-(4-nitrophenyl)-2H-5-tetrazolio]-1.3-benzene disulfonate (WST-1) kit (Roche, Mannheim, Germany). The colorimetric assay in viable cells depends on the mitochondrial dehydrogenases induced cleavage of the tetrazolium salt WST-1. WST-1 solution (100 µl/well) was added to cells in 6-well plates and subsequently incubated for one hour at 37 °C. The plate was recorded on a spectrophotometer at wavelength 440 nanometer (nm) with a reference at 690 nm.

**Trypan blue exclusion assay**

Trypan blue staining in conjunction with an automated cell viability analyzer was used (Vi CELL XR (Beckman Coulter, Inc., Brea, California, The USA) to evaluate cytotoxicity. VEGF enriched CVECs were plated at a density of 20,000 cells/well in 6-well plates, treated with escalating doses of pazopanib. At standardized interval, cells were trypsinized with 1 mL of Trypsin-EDTA (Invitrogen) for 3 min at 37ºC. In line with the manufacturer’s instructions, cells were resuspended in 500 µL growth media and tallied immediately using the ViCell XR Cell Proliferation Analyzer (Beckman-Coulter, Brea California, The USA). We documented automated cell proliferation tallies together with total number of cells.

**Measurement of Reactive oxygen species (ROS)**

Intracellular ROS was measured using dihydrorhodamine123 (AnaSpec EGT, Fremont, California, The USA), as a freely permeable non-fluorescent probe that identifies mitochondria, and fluoresces after oxidation by ROS. Cells were treated with 10 µM Dihydrorhodamine123. On excitation at 485 nm, rhodamine fluoresces at 528 nm, which was assessed applying multidetection microplate reader at separate time intervals.

**Intracellular analysis of caspase assay 3 activity**

The levels of activated caspase 3 were investigated at 72h, (a protease instrumental in the induction of apoptosis) after treatment with different doses of pazopanib. Activation of caspase 3 was recorded according to the manufacturer’s instructions (Thermo Scientific, Logan, UT, The USA). Briefly, following treatment of cells with variable doses of pazopanib, cells were fixed with 4% paraformaldehyde and permeabilized for 15 minutes using 31 surfactants X-100. Cell permeabilization was quenched with 30% hydrogen peroxide after that a blocking buffer was added for 30 minutes. Thereupon, cells were incubated with 1:500 dilution of anti–caspase 3 and a-tubulin (control), at 48 °C overnight. Then washed and were incubated for 30 minutes at ambient temperature with the anti-rabbit HRP conjugate. Afterwards, 3,3’,5,5’ Tetramethylbenzidine substrate was added. Cleaved caspase-3 protein levels were analyzed by grading the absorbance at 450 nm with microplate reader. We correlated the outcomes against a-tubulin control following detection with background absorbance and defined as percentage. Experiments were repeated three times to ensure repeatability of results. 

**Cellular morphology**

To observe the morphological changes, control cells, along with cells exposed to different doses of pazopanib were plated in 6 well plates. Each well was assessed independently under a microscope named as bright-field phase contrast IX51 (Olympus, Center Valley, Pennsylvania, The USA) at different time intervals. Cell architecture and morphology were re-examined after the cells were exposed to increasing doses of pazopanib. Signs of gross cellular injury e.g. variations in cytoplasmic or nuclear appearance as a result of cytotoxicity, were analyzed in both treated and control cells. For each dose studied, randomly selected photographs were taken at 20X magnification and evaluated.

**Statistical analysis**

Each experiment was repeated three times for each cell type at each pazopanib dose and time points. The mean ± standard deviation (SD) was reported. Statistical analysis was performed using ANOVA (Graphpad Prism, La Jolla Ca, The USA). P value<0.05 was considered as statistically significant.

## RESULTS

**VEGF enriched CVECs Cell Proliferation: WST-1 Assay**

**Figure 1:**
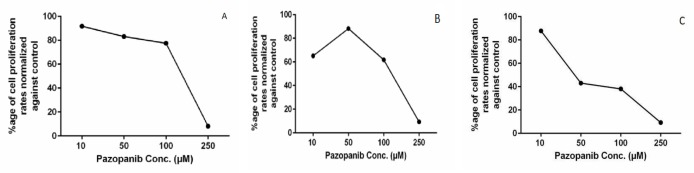
Effect of pazopanib (10, 50, 100, and 250 µM) on choroidal vascular endothelial cells (CVECs) enriched with vascular endothelial growth factor (VEGF: 50ng/mL). Cell proliferation was determined by WST-1 assay at different time intervals. Results expressed as percentage of cell proliferation compared to control. A. 48h, B. 72h, and, C. 1 Week. Abbreviations: µM: micromole; ng/mL: nanogram per millilitre; h: hour; %: percentage; Conc.: concentration.

The proliferation of CVECs in response to different concentrations of pazopanib at various time points is summarized in Table 1 and Figure 1. The growth rates were evaluated with WST-1 assay and described as percentage of surviving cells.

**Table 1:** Proliferation of CVECs in response to different concentrations of pazopanib at various time points. The proliferation rates were assessed using WST-1 assay.

Cell type / Incubation Time	Pazopanib Concentration (μM)			
CVECs	**10**	**50**	**100**	**250**
48h; mean ± SD	91.8%±4.74	83.1%±6.3	77.5%±1.87	8.2%±8.05
72h; mean ± SD	65.1%±19.8	88.2%±6.9	61.8%±13.4	9.3%±9.1
1Week; mean ± SD	87.8%±15.3	43.0%±6.7	38.1%±18.3	9.3%±24.9

Abbreviations: μM: micromolar; CVECs: choroidal vascular endothelial cells; h: hours; SD: standard deviation.

**48** **-** **hour Time point**

Treatment of VEGF enriched CVECs treated with various concentrations of pazopanib induced a decreased in cells-variability by 91.8% (p<0.001), 83.1% (p<0.001), 77.5% (p<0.001) and 8.2% (p<0.001) for pazopanib concentrations of 10, 50,100 and 250 µM, respectively compared to control cells (Table 1 and Figure 1).

**72** **-** **hour Time Point**

Treatment of VEGF enriched CVECs with pazopanib (10, 50,100 and 250 µM) concentrations resulted in a decrease in cell proliferation rates as 65.1% (p<0.001), 88.2% (p<0.001), 61.8% (p<0.001) and 9.3% (p<0.001) compared to control cells (Table 1 and Figure 1).

**One-week time point**

Based on WST-1 assay cell proliferation assay, at one-week time point, VEGF enriched CVECs also showed a decrease in cells proliferation rate at 87.8% (p<0.001), 43% (p<0.001), 38.1% (p<0.001) and 9.3% (p<0.001) respectively compared to control cells. (Table 1 and Figure 1).

**VEGF enriched CVECs Cell Proliferation: Trypan blue exclusion assay**

**Figure 2:**
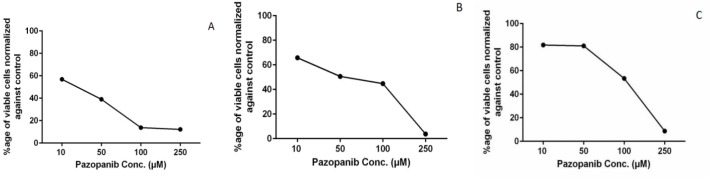
Effect of pazopanib (10, 50, 100, 250 µM) on choroidal vascular endothelial cells (CVECs) enriched with vascular endothelial growth factor (VEGF: 50ng/mL). Cell viability was defined by trypan blue assay using ViCell XR Cell analyzer at different time intervals. Results expressed as percentage of cell proliferation compared to control. A. 48h, B. 72h, and, C. 1 Week. Abbreviations: µM: micromole; ng/mL: nanogram per millilitre; h: hour; %: percentage; Conc.: concentration.

The percentage of viable cells of VEGF enriched CVECs in response to pazopanib treatment at different concentrations and various time points is summarized in Table 2. The cells viability rates were assessed with trypan blue exclusion assay as described in methods and presented as percentage of surviving cells (Figure 2).

**48-hour Time point**

Treatment of VEGF-enriched CVECs with pazopanib (10, 50,100 and 250 µM) showed decrease in viability by 56.9% (p=0.001), 39% (p=0.0001), 13.7% (p<0.0001) and 12.2% (p<0.0001) compared to controls (Table 2 and Figure 2).

**Table 2:** The percentage of viable cells of VEGF enriched CVECs in response to pazopanib treatment at different concentrations and various time points. The cells viability rates were assessed using trypan blue exclusion assay as described in methods.

Cell type / Incubation Time	Pazopanib Concentration (μM)			
CVECs	10	50	100	250
48h; mean ± SD	56.9%±29.5	39.0%±4.31	13.7%±15.1	12.2%±13.7
72h; mean ± SD	65.8%±11.3	50.61%±40.7	44.8%±14.4	3.8%±4.4
1Week; mean ± SD	81.8%±16.2	81.0%±16	53.4%±13.9	8.7%±6.1

Abbreviations: μM: micromolar; VEGF: vascular endothelial growth factor; CVECs: choroidal vascular endothelial cells; h: hours; SD: standard deviation.

72-hour Time Point

At 72-hour time point, VEGF-enriched CVECs showed a decline in cell viability after treatment with pazopanib by 65.8% (p=0.001), 50.61% (p<0.001), 44.8% (p<0.001) and 3.8% (p<0.001) as compared to control cells (Table 2 and Figure 2).

One-week time point

Based on trypan blue exclusion cell viability assay, at one-week time point, treatment of VEGF-enriched CVECs with pazopanib showed similar trend i.e. decrease in cell viability by 81.8% (p=0.24), 81% (p=0.21), 53.4% (p=0.001) and 8.7% (p<0.0001) as compared to control cells (Table 2 and Figure 2).

Intracellular reactive oxygen species levels after pazopanib treatment

**Figure 3:**
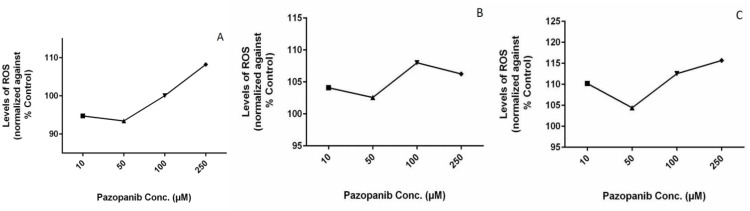
Reactive oxygen species (ROS) levels measured using dihydrorhodamine 123 (DHR 123) after exposure to various concentrations of pazopanib at different time intervals. A. 48h, B. 72h, and, C. 1 Week.

Abbreviations: µM: micromole; ng/mL: nanogram per millilitre; h: hour; %: percentage; Conc.: concentration.

Figure 3 summarizes the changes in the levels of ROS as a result of treatment of pazopanib at different concentrations and at different time intervals. At 48h, treatment of VEGF-enriched CVECs with pazopanib (10, 50,100 and 250 µM) resulted in 0.94%, 0.94%, 1% and 8 % increase in the ROS levels compared to controls. Similarly, at 72h ROS levels were increased by 4%, 2%, 8% and 6% compared to controls. Furthermore, at 1 week, ROS levels were increased by 10%, 4%, 12% and 15 % compared to controls. The results indicated a trend of increased levels of ROS by increasing pazopanib concentration. However, the increased difference between exposed cells and the control cells were not statistically significant.

**Figure 4:**
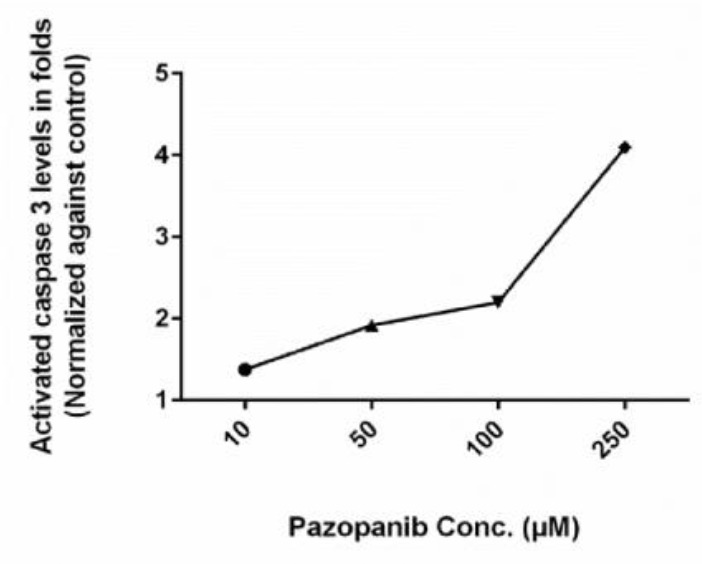
Measurement of activated caspase-3 levels in CVECs enriched with VEGF treated with different concentrations of pazopanib at 72h showed a 5-fold increase in activated caspase 3 levels compared to control. Abbreviations: µM: micromole; h: hour; VEGF: vascular endothelial growth factor; CVECs: choroidal vascular endothelial cells; Conc.: concentration.

Activated Caspase 3 Evaluation:

Treatment of VEGF enriched CVECs with varying doses of pazopanib (10, 50,100 and 250 µM) resulted in a dose dependent increase in the release of caspase-3 from cells. At 72h, 1.38, 1.92, 1.92 and 4.1 fold increase (p<0.001) in the caspase-3 levels were observed compared to controls (Figure 4). These results suggest that pazopanib actively inhibits the proliferation of VEGF enriched CVECs through promotion of apoptosis.

Morphology of VEGF enriched CVECs

**Figure 5:**
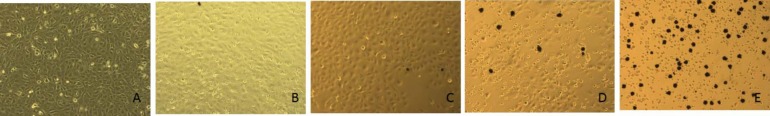
Effect of different concentrations of pazopanib on cell morphology of VEGF enriched choroidal vascular endothelial cells at 72 hrs; Decrease in cell size and irregular membrane was observed compared to controls. Bright field images were taken at 20X magnification (A‐E: Control, 10, 50, 100 and 250 micrometer).

Cellular changes after pazopanib treatments (10, 50,100 and 250 µM) were assessed by bright field microscopy (Olympus Corporation, Tokyo, Japan) at 48h. Representative photographs are presented in Figure 5. Bright field microscopy of VEGF enriched CVECs in culture, revealed a decrease in cell size and irregular membrane structure after exposure to high pazopanib concentration of 250 µM.

## Discussion

Our study confirmed that VEGF-enriched CVECs treated with escalating doses of pazopanib decreased cell viability and increased caspase 3 levels in a time and dose dependent manner. Since, AMD is the foremost etiology of blindness in the elderly in developed countries, we believe that our findings supplement the ongoing efforts in development of new pharmacologic and therapeutic measures.

Often vision loss caused by AMD is attributed to neovascular form of the disease [1]. Anti-angiogenic drugs that target VEGF are very effective in treating exudative AMD and currently used as a standard of care in management of active AMD. However, they need multiple intravitreal injections, usually monthly. Hypothetically, the immature proliferating CVECs are a target for anti-angiogenic agents to inhibit proliferation. Although few clinical investigations evaluated the therapeutic action of pazopanib in the treatment of CNV, there is no preclinical data that examined the dose of pazopanib necessary for inhibition of the CVEC proliferation [19]**.** In addition, poor therapeutic response was observed at the doses tested in the clinical trial [19]. In this study, we investigated and found a dose-dependent inhibition of proliferating CVECs after exposure to Pazopanib, a multi-targeted tyrosine kinase inhibitor of VEGF receptors (VEGFRs)-1, VEGFRs-2 and VEGFRs-3, stem cell growth factor C-Kit-17 and PDGFRs-α and β. This small molecule is a potent antiangiogenic molecule and FDA approved biological agent used in the treatment of soft tissue sarcoma and advanced renal cell carcinoma. 

Pazopanib in eye drop (5mg/mL) and oral formulations has been investigated for medical treatment of neovascular AMD [19-22]. However, topical eye drops were clinically ineffective. Pazopanib may inhibit the similar pathway clinically substantiated and confirmed in AMD with anti-VEGF therapies, but through inhibition of tyrosine kinase receptor. Added benefit may derive from inhibition of the pro-angiogenic PDGF pathway. PDGF stabilizes new vessels and basement membrane and enhances resistance to anti-VEFG agents. Inhibition of PDGFR-α and PDGFR-β, in conjunction to inhibition of the VEGF pathway, could lead to lasting CNV regression. To the best of our knowledge this is the first report establishing the inhibitory effects of escalating doses of pazopanib on proliferation of VEGF-enriched CVECs. Pazopanib, a multi-targeted tyrosine kinase inhibitor of VEGF receptors promotes cell damage and death. In the current study, reduction in cell viability positively correlated with an increase in dose at different time intervals. This suggests that pazopanib induces vascular cell death through the blockage of tyrosine kinase (that was sustained days) following treatment.

Sequential increase in cell death with increasing concentration of pazopanib was observed with WST-1 assay. There was variable response in cell viability with 10 and 50 µM concentrations of pazopanib. There was a linear decrease in cell viability with increase in time (p<0.001) at 100 µM concentration. At 250 µM concentration, toxic effect of pazopanib was noted, with death of 91% of cells (both at 48 hours and 1 week time points). Contrary to the results of WST-1, the results of trypan blue exclusion assay showed an increase in cell viability over the course of time at 10, 50 and 100 µM concentrations. Maximum cell death was noted after 48 hours of exposure. However, the number of dead cells decreased at 72 hours or 1 week.

At 250 µM concentration, the toxic effect of pazopanib was noted at all the time points. Similar to WST-1, there was an increase in cell death with increased concentration of pazopanib. This difference in response between the results of the two methods is probably secondary to dissimilar underlying mechanisms. The property of trypan blue exclusion assay is staining of dead cells and injured cell membrane while the basis of WST-1 assay is the enzymatic cleavage of the tetrazolium salt WST-1 by cellular mitochondrial dehydrogenases available in viable cells. WST-1 produces a mass response (quantitative data), whereas trypan blue method provides a response from the individual cell (qualitative data). While the trypan blue exclusion assay may have higher specificity for cell death detection, the WST-1 assay has a higher sensitivity.

Morphologic changes showed a decrease in cells size and irregular membrane structure and this was more evident at high pazopanib concentration of 250 µM (toxic effect of pazopanib at higher concentration). ROS generated from the interaction of cell membrane lipids, are known as cell damage and death inducer. There was an increase in ROS compared to the controls with exposure to pazopanib, though not statistically significant. Similarly, there was a gradual increase in the levels of caspase-3 on treatment with varying doses of Pazopanib and an exponential increase was observed at 250 µM concentration compared to controls, suggesting that pazopanib actively inhibits the proliferation of VEGF enriched CVECs and further promotes apoptosis.

Danis et al. reported the applicability of eye drop formulation of pazopanib in patients with CNV. They noted significant improvement in mean vision at 1 month [19]. However, improvement in macular edema was found only in the subset of subjects [CFH Y402H TT genotype]. Csaky et al. In a study on patients with AMD who had previously received intravitreal injections evaluated the effect of daily pazopanib eye drops (5mg/mL QID) on the stability or improvement of visual acuity, along with a decrease in the continuous need for intravitreal injections [23]. They also evaluated the safety, tolerability and changes in the retina associated with this eye drop and determined the steady-state plasma concentration of the drug after administration of the eye drop. They concluded that the eye pazopanib eye drops did not reduce the frequency of as-needed injections. Pazopanib eye drop did not show increased efficacy in this study because of insufficient transduction of active drug to the relevant ocular tissues (retina/choroid). However, the drop, administered four times daily for 52 weeks, was well tolerated by patients. Its ophthalmic and non-ocular complications were also mild and no drug-related severe adverse effects were reported. 

Takahashi et al. reported regression of CNV in mouse model after oral administration of pazopanib [24]. Systemically administered drugs have their own disadvantages. Usually lower amount of drug reaches the target tissue, large portion of the drug is destroyed metabolically before it reaches target tissue and there are unavoidable systemic side effects. Intravitreal injection of pazopanib is more likely to be effective therapeutically, as it provides necessary dose directly to the target tissue. Before the use of intravitreal pazopanib, its toxicity profile on choroidal and RPE cells needs to be evaluated.

The main strengths of our current study include the reproducibility of the CVEC model, and reliability of the data through multiple methods including WST 1 assay, trypan blue assay, ROS levels and caspase essay performed in triplicates. The main limitation of the study was its *in vitro* nature. We believe that replication of these results through an animal model would validate the conclusions of our study and establish a dose that is required for inhibition of neovascular membrane with intravitreal injection of pazopanib.

## CONCLUSION

In conclusion, our study showed that VEGF enriched proliferative CVECs are particularly sensitive to Pazopanib in a dose dependent manner and cell death results from inhibition of tyrosine kinase. Topical drops could be formulated with an intent to increase intravitreal concentration of Pazopanib in the range of 250µM for therapeutic effect.

## References

[1] Gehrs KM, Anderson DH, Johnson LV, Hageman GS (2006). Age-related macular degeneration--emerging pathogenetic and therapeutic concepts. Ann Med.

[2] Congdon N, O'Colmain B, Klaver CC, Klein R, Munoz B, Friedman DS (2004). Causes and prevalence of visual impairment among adults in the United States. Arch Ophthalmol.

[3] Chalam KV, Balaiya S, Malyappa RS, Hsi W, Brar VS, Murthy RK (2011). Evaluation of choroidal endothelial cell proliferation after exposure to varying doses of proton beam radiation. Retina.

[4] (1999). Photodynamic therapy of subfoveal choroidal neovascularization in age-related macular degeneration with verteporfin: one-year results of 2 randomized clinical trials--TAP report Treatment of age-related macular degeneration with photodynamic therapy (TAP) Study Group. Arch Ophthalmol.

[5] Isola V, Pece A, Parodi MB (2006). Choroidal ischemia after photodynamic therapy with verteporfin for choroidal neovascularization. Am J Ophthalmol.

[6] Mynampati BK, Sambhav K, Grover S, Chalam KV (2017). Inhibition of proliferation of retinal vascular endothelial cells more effectively than choroidal vascular endothelial cell proliferation by bevacizumab. Int J Ophthalmol.

[7] Bhutto I, Lutty G (2012). Understanding age-related macular degeneration (AMD): relationships between the photoreceptor/retinal pigment epithelium/Bruch's membrane/choriocapillaris complex. Mol Aspects Med.

[8] Mohan N, Monickaraj F, Balasubramanyam M, Rema M, Mohan V (2012). Imbalanced levels of angiogenic and angiostatic factors in vitreous, plasma and postmortem retinal tissue of patients with proliferative diabetic retinopathy. J Diabetes Complications.

[9] Kim SY, Mocanu C, McLeod DS, Bhutto IA, Merges C, Eid M (2003). Expression of pigment epithelium-derived factor (PEDF) and vascular endothelial growth factor (VEGF) in sickle cell retina and choroid. Experimental Eye Research.

[10] Vasudev NS, Reynolds AR (2014). Anti-angiogenic therapy for cancer: current progress, unresolved questions and future directions. Angiogenesis.

[11] Zhou J, Shukla VV, John D, Chen C (2015). Human Milk Feeding as a Protective Factor for Retinopathy of Prematurity: A Meta-analysis. Pediatrics.

[12] Yang S, Zhao J, Sun X (2016). Resistance to anti-VEGF therapy in neovascular age-related macular degeneration: a comprehensive review. Drug Des Devel Ther.

[13] Boyer DS, Hopkins JJ, Sorof J, Ehrlich JS (2013). Anti-vascular endothelial growth factor therapy for diabetic macular edema. Ther Adv Endocrinol Metab.

[14] Al-Khersan H, Hussain RM, Ciulla TA, Dugel PU (2019). Innovative therapies for neovascular age-related macular degeneration. Expert Opin Pharmacother.

[15] Barakat MR, Kaiser PK (2009). VEGF inhibitors for the treatment of neovascular age-related macular degeneration. Expert Opin Investig Drugs.

[16] Chappelow AV, Kaiser PK (2008). Neovascular age-related macular degeneration: potential therapies. Drugs.

[17] Mousa SA, Mousa SS (2010). Current status of vascular endothelial growth factor inhibition in age-related macular degeneration. BioDrugs.

[18] Ni Z, Hui P (2009). Emerging pharmacologic therapies for wet age-related macular degeneration. Ophthalmologica.

[19] Danis R, McLaughlin MM, Tolentino M, Staurenghi G, Ye L, Xu CF (2014). Pazopanib eye drops: a randomised trial in neovascular age-related macular degeneration. Br J Ophthalmol.

[20] Suda K, Murakami T, Gotoh N, Fukuda R, Hashida Y, Hashida M (2017). High-density lipoprotein mutant eye drops for the treatment of posterior eye diseases. J Control Release.

[21] Singh R, Wurzelmann JI, Ye L, Henderson L, Hossain M, Trivedi T, Kelly DS (2014). Clinical evaluation of pazopanib eye drops in healthy subjects and in subjects with neovascular age-related macular degeneration. Retina.

[22] McLaughlin MM, Paglione MG, Slakter J, Tolentino M, Ye L, Xu CF (2013). Initial exploration of oral pazopanib in healthy participants and patients with age-related macular degeneration. JAMA Ophthalmol.

[23] Csaky KG, Dugel PU, Pierce AJ, Fries MA, Kelly DS, Danis RP (2015). Clinical evaluation of pazopanib eye drops versus ranibizumab intravitreal injections in subjects with neovascular age-related macular degeneration. Ophthalmology.

[24] Takahashi K, Saishin Y, Saishin Y, King AG, Levin R, Campochiaro PA (2009). Suppression and regression of choroidal neovascularization by the multitargeted kinase inhibitor pazopanib. Arch Ophthalmol.

